# Quality evaluation of processed clay soil samples

**DOI:** 10.11604/pamj.2016.24.118.8406

**Published:** 2016-06-06

**Authors:** Matilda Steiner-Asiedu, Obed Akwaa Harrison, Frederick Vuvor, Kwaku Tano-Debrah

**Affiliations:** 1 Department of Nutrition and Food Science, School of Biological Sciences, College of Basic and Applied Sciences, University of Ghana, Legon, Ghana

**Keywords:** Pica, geophagia, clay, ayilo, microbiology

## Abstract

**Introduction:**

This study assessed the microbial quality of clay samples sold on two of the major Ghanaian markets.

**Methods:**

The study was a cross-sectional assessing the evaluation of processed clay and effects it has on the nutrition of the consumers in the political capital town of Ghana. The items for the examination was processed clay soil samples.

**Results:**

Staphylococcus spp and fecal coliforms including Klebsiella, Escherichia, and Shigella and Enterobacterspp were isolated from the clay samples. Samples from the Kaneshie market in Accra recorded the highest total viable counts 6.5 Log cfu/g and Staphylococcal count 5.8 Log cfu/g. For fecal coliforms, Madina market samples had the highest count 6.5 Log cfu/g and also recorded the highest levels of yeast and mould. For Koforidua, total viable count was highest in the samples from the Zongo market 6.3 Log cfu/g. Central market samples had the highest count of fecal coliforms 4.6 Log cfu/g and yeasts and moulds 6.5 Log cfu/g. “Small” market recorded the highest staphylococcal count 6.2 Log cfu/g. The water activity of the clay samples were low, and ranged between 0.65±0.01 and 0.66±0.00 for samples collected from Koforidua and Accra respectively.

**Conclusion:**

The clay samples were found to contain Klebsiella spp. Escherichia, Enterobacter, Shigella spp. staphylococcus spp., yeast and mould. These have health implications when consumed.

## Introduction

The consumption of nonfood materials is a common practice in many cultures in different parts of the world. Pica is the broad term that describes this habit and it has been defined as the pathological act of eating nonfood items [1]. A common example of pica material is clay soil. Pica is categorized based on the type of material eaten. The eating of clay soil is termed as geophagia; amylophagia is for the consumption of cornstarch, while Pagophagia describes the consumption of ice [2]. Other pica substances are wall board, chalk, charcoal and even toilet paper however pagophagia does not carry the same risk as geophagia because it serves as an alternative means of hydration [2]. The global prevalence of geophagia has been confirmed while the incidence of pica (of which geophagia is a form) is not limited to any one geographic area, race, sex, culture, or social status, nor is it limited to pregnancy [2]. Studies have also shown that age is also not a limitation to geophagia since it's a habitude in children and adults [3]. In Ghana geophagia is highly prevalent among women and children. The prevalence of clay eating among Ghanaians has been found to be 17% and 22% in pregnant and lactating women respectively [4]. Also among 502 women attending antenatal clinics in Accra, a prevalence of 28% was observed [5]. The daily consumption of clay material has been reported to be between the ranges of 3.5 to 488g, with an estimated average of 70g per person [5]. Clays are soil materials and could be a natural habitat of microorganisms which may involve parasitic and pathogenic species [6]. The isolation of coliform bacteria in dry white clay samples from 3 major markets in Accra confirms the above observation [6].

Traditionally, clay is used for making drinking and cooking pots with the belief that it has the ability to bind toxins. In Ghana, edible clay is called “eshire” by the Akan tribes, “farankese” by some northern tribes, “fefe” by the Ewes while the Gas call it “ayilo”. The clay soil is dug from the earth and prepared by milling the large particles and blocks to help reduce the particle size. The fine powder is then mixed with water and stirred into a uniform paste. Afterwards, the paste is molded into a sausage- like shape and then baked to reduce the moisture content and retain the shape. In some cultures the molded paste is baked and smoked to give it an appealing aroma. Both types are found commonly on the Ghanaian market. Geophagia is very prevalent among pregnant woman who are believed to develop craving for clay as a remedy for morning sickness and vomiting [4]. This trend must be critically looked at because it has serious health implications. For example, in Ghana maternal anemia is highly prevalent and a documentary on Maternal Health Channel showed that pregnant women do consume a lot of these clay substances which may bind and prevent iron absorption [7]. Although there have been some studies on pica in pregnant Ghanaians [5], on Geophagia among lactating and pregnant women [4], and on the microbiology of dry white clay [6], these have not been exhaustive enough to cover the general population on issues regarding people's knowledge and perceptions concerning geophagia and microbial load of clay products on the Ghanaian market. This study therefore seeks to identify and characterize any possible genera of microorganisms in clay products. The health implications of the consumption of clay may be evident and this may form the basis for educational campaigns to promote nutrition and health.

## Methods

**Sample collection:** This study involved laboratory analysis of clay samples. Also, a survey was done in the selected markets to identify the types of clay that are sold. Microbiological analysis was carried out on the clay samples that were purchased from the selected major markets in Accra and Koforidua. The markets selected in Accra were; Madina, Makola and Kaneshie markets. For Koforidua, Zongo market, Dwakesiem (Central market) and Dwaketoam (Small market) were selected. These represented the Greater Accra and Eastern Regions of Ghana. In each of the markets, the samples were bought from different retailers. A quick market survey was done at the selected major markets in Accra (namely; Madina, Makola, and Kaneshie markets) to identify the different types of clay samples sold on the market. The various types of clay samples available on the markets were examined by looking at the differences in the shapes, colors, sizes and their purpose. The simple random sampling method was used for both the survey and laboratory analysis. The clay samples were collected with stomacher bags and sealed with cello tape and properly labeled with the market it was bought from. Samples were handled with care and treated under aseptic conditions to avoid any contamination. At each market, 20 pieces of the clay were purchased randomly from different retailers and packaged into sterile stomacher bags. The stomacher bags were then carefully sealed with cello tape and labeled appropriately by the names of the specific markets they were bought from.

**Microbiological analyses:** The bacteria yeast and molds present in the samples were enumerated using specific nutrient media. Series of morphological examinations and biochemical tests were conducted to help characterize and identify the specific microorganisms that were growing on the clay samples. Details of these procedures are described as follows; dehydrated media (Oxoid and Difco Limited, Basingstoke, Hampshire England) were used in the analysis. The media used were: Nutrient Agar (NA) (to help in the acquisition of a pure culture), Levine Eosin Methylene Blue (EMB) (to determine the presence of coliforms), Mannitol Salt Agar (MSA) (to detect staphylococcus species), Plate Count Agar (PCA) (for total viable count), Yeast Extract Agar (YEA) (for detection of yeasts and molds), and Peptone Water (used as a diluent). They were prepared according to the manufacturer's instructions. For enumeration and characterization of microorganisms, samples from each market were aseptically homogenized by crushing and about 10 grams aseptically transferred into new stomacher bags and blended with 90 ml of sterile diluent (maximum recovery diluents), in a Seward stomacher blender 80 (Seward company limited, England) for 1 minute. Aliquots of the homogenates were serially diluted and plated for the determination of total plate count and the detection of coliforms, staphylococcal spp. and fungi, using standard procedures. For the identification and characterization of the microorganisms in the samples, some morphological and biochemical examinations were done as described by Prescott, Harley and Klein [8]. These included: Gram Staining, Catalase test, Peroxidase test, Triple Sugar Iron (TSI) agar test, Sulphur Indole Motility (SIM) test and the Simmons Citrate test.

### Physicochemical parameters of clay soil samples

**Water holding capacity:** An amount of 5 grams of the powdered sample was weighed into a centrifuge tube. 30 ml of water was added and mixed using the vortex mixer for 30 seconds. The mixture was made to stand for 30 minutes and stirred after every 10 minutes until the 30 minutes was due. This was followed by centrifugation using a bench top centrifuge set at 3000 rpm for 15 minutes. The supernatant was decanted and the weight of the residue was recorded. The amount of water absorbed, expressed as a percentage of the dry sample is equivalent to the gram of water per gram of sample that it could hold. Triplicate measurement was taken for each sample.

Percent water holding capacity = weight of hydrated sample-weight of sample X 100 weight of dry sample

**Water activity:** The water activity of the clay samples was determined using the Retronic hycropalm water activity meter (Retronic AG company limited, model number: HP23-AW-A). An amount of 2 grams of the powdered sample was weighed into clean dishes. The dish was then placed into the panel of the water activity meter. The probe was then used to cover the dish containing the sample. The start button of the meter was pressed and the water activity reading was recorded after about 2 minutes when the reading had stabilized. This was done in triplicates for each sample and the average was taken.

**Moisture content:** The air-oven method was used for this determination. The temperature of the oven was adjusted to 105 °C0 C to precondition the moisture cans for about 15 minutes. Afterwards, the moisture cans were removed and made to cool in a desiccator. Approximately 2 g of the powdered clay samples was weighed into the cans and were transferred into the oven. The sample was left overnight to allow the moisture in it to be evaporated. After 24 hours the final weight of the sample was taken and the moisture content of the sample was calculated on dry mater basis. This was repeated thrice for each of the samples average was taken.

Percent Moisture content = weight of hydrated sample-weight of sample X 100 weight of dry sample

**Data processing and analysis:** For microbiological analysis, the data was entered into Microsoft Excel (2010). Means and standard deviations were computed and graphical representations were used where appropriate.

## Results

**Figure 1:**
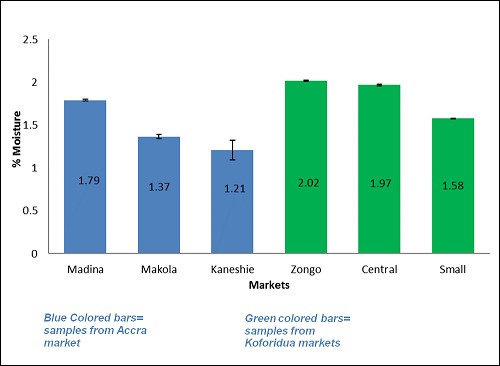
Moisture content of clay sample

**Figure 2:**
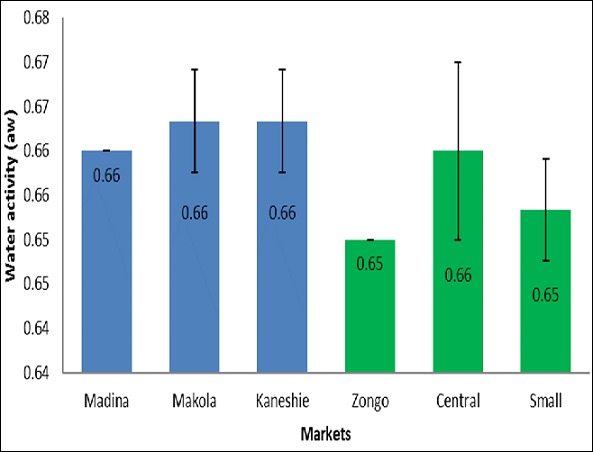
Water activity of the clay samples

**Figure 3:**
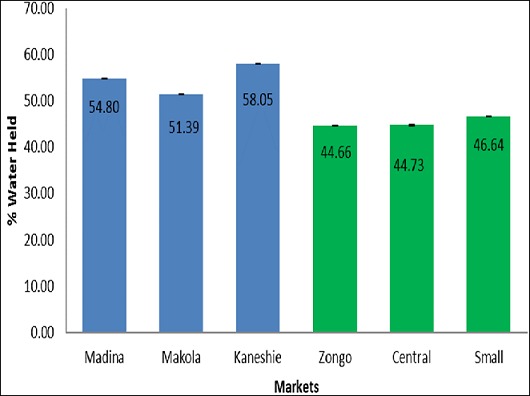
Water holding capacity of clay samples

Table 1 shows the various types of clay samples available on the market. In all 7 different types of clay samples were found to be sold on the market. They varied in shapes, sizes and colors. The weights of the clay samples ranged from 8.99 grams to 252.55 grams. Table 2 shows the total viable counts, coliform counts, staphylococcal counts, and yeast and moulds counts in the samples from the selected markets in Accra and Koforidua. Samples from the Kaneshie market in Accra recorded the highest total viable counts 6.5 Log cfu/g. For fecal coliforms, Madina market samples had the highest count 6.5 Log cfu/g. Again, samples from the Madina market were contaminated with yeast and mould the most. Staphylococcal count was highest in the samples from the Kaneshie market in Accra 5.8 Log cfu/g. For Koforidua, total viable count was highest in the samples from the Zongo market 6.3 Log cfu/g. The samples from the Central market had the highest count of fecal coliforms 4.6 Log cfu/g and yeasts and moulds 6.5 Log cfu/g. small market recorded the highest staphylococcal count 6.2 Log cfu/g. Figure 1 illustrates the moisture contents of clay samples from the selected markets in Accra (shown in blue bars) and Koforidua (shown in green bars). The samples from Accra had an average moisture content of 1.46±0.30%, with samples from Madina market recording the highest moisture of 1.79±0.01%. The average moisture in the samples from Koforidua markets was 1.86±0.24%. In koforidua, samples from the Zongo market recorded the highest moisture content of 2.02±0.01%. Figure 2 displays the water activity of clay samples from the selected markets in Accra (shown in blue bars) and Koforidua (shown in green bars). The Accra samples recorded a mean water activity of 0.66±0.00, with those samples from Makola and Kaneshie markets having the same moisture content of 0.66±0.01. The samples from Koforidua had an average water activity of 0.65±0.01, with those from central market recording the highest water activity 0.66±0.01. Figure 3 illustrates the water holding activity of clay samples from the selected markets in Accra (shown in blue bars) and Koforidua (shown in green bars). The average percentage of water held in the samples from Accra 54.75±3.33%, with samples from Makola market recording the highest amount of water held 58.05±0.01%. The Koforidua samples however recorded a lower average amount of water held 45.34±1.12%, with the sample with the highest percent water held coming from small market 46.64±0.02%.

**Table 1:** Types of clay samples available on the market (N=7)

TYPES	SHAPE	COLOUR	WEIGHT (g)
Model i	Rectangular	yellowish-grey	8.99
Model ii	Oval	Dark ash	9.22
Model iii	Oval	White	9.22
Model iv	oval	white	42.23
Model v	sausage-like	white	252.55
Model vi	circular	yellowish-grey	125.56
Model vii	round	yellowish-grey	159.48

**Table 2:** Microbial content of clay from different locations

Source (Market)	Total Viable Count (Log cfu/g)	Fecal Coliform Count (Log cfu/g)	Yeast & Moulds Count (Log cfu/g)	Staphylococcal Count (Log cfu/g)
**Accra**				
Madina	6.4	6.5	6.3	5.7
Makola	6.4	6.4	16.2	5.1
Kaneshie	6.5	6.4	6.1	5.8
**Koforidua**				
Zongo	6.3	NIL	6.3	6.0
Central	6.2	4.6	6.5	6.1
Small	6.1	4.3	6.4	6.2

## Discussion

The clay samples from both Accra and Koforidua had lower moisture content thus 1.46±0.30% for the Accra samples and 1.86±0.24% for the Koforidua samples. The water activity of the samples were also low thus 0.66±0.00 and 0.65±0.01 for the Accra and Koforidua samples respectively. In another study, the moisture content of clay samples was observed to be between 0.18-0.19% when the air oven method was used [9]. These values show that the clay samples were too dry to support microbial growth. Microorganisms require high water activities to grow. Most bacteria including clostridium botulinum do not grow at water activities below 0.91, and at water activities below 0.60; no microbiological growth is possible [6]. The water activities of the samples were a little above 0.60, which means some microbial growth could be found on the clay lumps. Water holding capacity tells the total amount of water a soil can hold at field capacity and also indirectly indicates the extent to which the internal part of material is aerated. The water holding capacity of normal clay that is not heat processed is quite high (thus 68%) and this increases with increasing content of clay in the soil [10]. The values recorded in this study are quite low especially in the samples from Koforidua, suggesting that the samples are poorly porous and may be unsuitable habitats for bacteria. The results of the microbiological analysis suggest that the the clay soil samples were contaminated by human waste or water which is likely to occur during post-processing handling. The detection of Klebsiella, Escherichia, Enterobacter and Shigella spp. was not surprising since these organisms are generally found in soil and water, or exist as saprophytic inhabitants of vertebrate intestinal tract that regularly contaminate soils [6]. Their presence in the clay may thus be by direct contamination from soil or fecal contamination due to the unhygienic handling of the products or unsanitary conditions under which the clays are processed and distributed. The frequency of isolating these organisms from plates prepared from the samples of different sources varied. The presence of Klebsiella, Escherichia, and Shigella and Enterobacter spp. in the samples could pose some risks to consumers [8]. As enteric microorganisms, their presence may also suggest the presence of other fecal coliforms which may be pathogenic.

Another genus of bacteria that were detected was staphylococcus spp. The detection of Staphylococcus spp also pointed to the unhygienic handling practices. Species of Staphylococcus are known food toxicants. Their presence could therefore implicate that there was some cross- contamination of the clay samples. Most spoilage bacteria, yeasts and molds require a minimum water activity of 0.9, 0.88 and 0.8 respectively [11]. The yeasts and moulds present in the samples could therefore possibly be xerophilic yeasts and moulds since they require a water activity of 0.66 [12]. Variations in microbial counts observed could be due to general hygiene observed among processors and sellers. Ready-to-eat foods that are cooked or roasted with further handling or processing before consumption are declared safe for consumption when total aerobic plate count, coliforms and yeasts and moulds are less than 1×107, 1×103 and 1×103 respectively [13]. This implies that the plate counts for the fecal coliforms was within the standards, however those for viable bacteria, yeast and molds far exceeded the limits and as such could pose risk to consumers. Pica can cause a number of nutritional and health problems and the complications vary depending on the type of pica (thus geophagia, amylophagia, pagophagia). The potential complications of pica include; nutrient deficiencies, constipation, electrolyte imbalances, gastrointestinal disturbances, parasitic infection, lead poisoning and associated complications, dental complications, weight gain, gestational hyperglycemia and metabolic disturbances [4]. Geophagia has numerous potential side effects as clay or soil can replace nutritive sources of food.

Hence, pica patients consume a less varied diet and are at risk of iron deficiency anemia. Additionally, kaolin which is a common ingredient in medications to treat diarrhea, may cause constipation in high amounts. Geophagia may cause hyperkalemia because clay can bind to potassium in the intestine and lead to increased excretion. The most dangerous of the complications of Geophagia is probably lead poisoning [4]. Lead exposure can lead to kidney damage, encephalopathy and impaired cognitive function in both the mother and the fetus [14]. The negative health effects of clay (ayilo) eating need greater public awareness which can be achieved through health talks both on TV and radio as well as during antenatal clinic visits. This is because all indications among the general public [15] and several prevalence studies [3–5] reveal that prevalence of consumption particularly among pregnant women and the general populace is on the rise. If behavior change continues to be a challenge with respect to abstinence, then there will be the need for an alternate new product with similar organoleptic characteristics be developed and possibly fortified with essential nutrients to at least meet some of the micronutrient needs of especially pregnant women who usually fall prey to this product due to pregnancy cravings. Furthermore, targeting the nursing professional with proper educations on the problems associated with the consumption of the clay will be a step in the right direction if the goal of improving maternal and child health within the 1st 10000 days should be recognized.

## Conclusion

The clay samples were found to contain Klebsiella spp. Escherichia, Enterobacter, Shigella spp. staphylococcus spp. yeast and mould. These findings calls for health promotion educational campaigns to halt consumptions of ayilo and related materials as this habit is detrimental to the health of particularly pregnant women and their unborn babies.

### What is known about this topic

- The prevalence of geophagia has been studied largely and has been found to be highest among pregnant women than the non-pregnant women and men;
- Studies have shown that these clay samples consumed could lead to heavy metal contamination and lead to micronutrient deficiencies;
- The microbiology of these processed clay soil samples have been done in some markets in Accra, Ghana.

### What this study adds

- This study further validated the microbiological quality of the clay samples in Greater Accra region and extended it to the Eastern region of Ghana;
- The different types (shapes, color, etc.) of clay samples available on the market was surveyed as well as the uses of these different types;
- Also the weight of the clay samples were determined in the Accra market to have an idea of how much clay can be consumed.
